# 1439. Epidemiology and 12- Month Antibiotic Use in the Outpatient Setting among Adult Patients with Complicated Urinary Tract Infections: A Retrospective Database Analysis

**DOI:** 10.1093/ofid/ofab466.1631

**Published:** 2021-12-04

**Authors:** Thomas Lodise, Thomas Lodise, Janna Manjelievskaia, Matthew Brouillette, Kate Sulham

**Affiliations:** 1 Albany College of Pharmacy and Health Sciences, Albany, NY; 2 IBM Watson Health, Cambridge, Massachusetts; 3 Spero Therapeutics, Cambridge, MA

## Abstract

**Background:**

Complicated urinary tract infections (cUTI) are one of the most common bacterial infections and represent substantial burden to the health care system. Here, we examine the epidemiology and treatment patterns associated with cUTI in a large US database containing longitudinal inpatient (IP) and outpatient (OP) patient-level data.

**Methods:**

We conducted a retrospective cohort study of adult patients in the IBM MarketScan® Commercial or Medicare Supplemental Databases with at least 1 IP or non-diagnostic OP claim with a diagnosis for cUTI between January 1, 2017 and June 30, 2019. Patients meeting the following criteria were included for analysis: (1) ≥18 years of age on the index date, (2) ≥6 months of continuous enrollment (CE) with medical and pharmacy benefits prior to the index date, (3) ≥12 months of CE following the index date or evidence of death, and (4) no evidence of a prior cUTI during the 6-month baseline period. Demographics and clinical characteristics were quantified. Patients were classified as IP if they were hospitalized during 30-day post index date; remaining patients were classified as OP. Antibiotics received in the OP setting in the 12-months post index date were examined.

**Results:**

95,423 patients met study criteria. Most (86.4%) patients were Commercially insured, mean (SD) age was 53.6 (18.1) and 70.4% were female. Mean baseline Charlson Comorbidity Index was 0.77. During the 30-day post index date, 22.2% were treated as IP and 77.8% were strictly treated as OP. In the 12-month OP follow-up period among index IP, 78.2% required ≥ 2 antibiotics, 38.2% required ≥4 antibiotics, and 41.6% received an IV antibiotic. In the 12-month OP follow-up period among index OP, 81.8% required ≥2 antibiotics, 38.2% required ≥4 antibiotics, and 46.8% received an IV antibiotic. For both IP and OP, fluoroquinolones were the most common oral antibiotic class (57.7%), followed by cephalosporins (39.2%), penicillins (30.3%), trimethoprim-sulfamethoxazole (29.8%), and nitrofurantoin (25.2%). Cephalosporins were the most common IV antibiotic class (38.5%).

**Conclusion:**

Regardless of index treatment setting, approximately 40% of all cUTI patients required ≥4 antibiotic therapy and almost half with receive an IV antibiotic in the outpatient setting in the 12-months post index date.

**Disclosures:**

**Thomas Lodise, Jr., PharmD, PhD**, **Astra-Zeneca** (Consultant)**Bayer** (Consultant)**DoseMe** (Consultant, Advisor or Review Panel member)**ferring** (Consultant)**genentech** (Consultant)**GSK** (Consultant)**Melinta** (Consultant)**merck** (Consultant, Independent Contractor)**nabriva** (Consultant)**paratek** (Consultant, Advisor or Review Panel member, Speaker’s Bureau)**shionogi** (Consultant, Advisor or Review Panel member, Speaker’s Bureau)**Spero** (Consultant)**tetraphase** (Consultant)**Venatrox** (Consultant) **Thomas Lodise, Jr., PharmD, PhD**, Melinta Therapeutics (Individual(s) Involved: Self): Consultant; Merck (Individual(s) Involved: Self): Consultant, Scientific Research Study Investigator; Paratek (Individual(s) Involved: Self): Consultant; Shionogi (Individual(s) Involved: Self): Consultant, Speakers’ bureau; Spero (Individual(s) Involved: Self): Consultant; Tetraphase Pharmaceuticals Inc. (Individual(s) Involved: Self): Consultant **Janna Manjelievskaia, PhD, MPH**, **IBM Watson Health** (Employee)**Spero Therapeutics** (Consultant) **Matthew Brouillette, MPH**, **Spero Therapeutics, Inc.** (Other Financial or Material Support, I am an employee of IBM Watson Health, who received funds from Spero Therapeutics, Inc. to conduct this analysis.) **Kate Sulham, MPH**, **Spero Therapeutics** (Consultant)

